# Slider Sheet Detection in Charge-Induction Electrostatic Film Actuators

**DOI:** 10.3390/s23031529

**Published:** 2023-01-30

**Authors:** Motoki Kojima, Shunsuke Yoshimoto, Akio Yamamoto

**Affiliations:** Department of Human and Engineered Environmental Studies, The University of Tokyo, Chiba 277-8563, Japan

**Keywords:** electrostatic motor, electrostatic actuator, linear motor, proximity sensing, built-in sensing

## Abstract

This work analyzes a built-in slider detection method for a charge-induction type electrostatic film actuator with a high surface-resistance slider. In the detection method, one stator electrode is detached from the parallel driving electrodes and is dedicated to sensing. When a slider with induced charges moves over the sensing electrode, electrostatic induction occurs in the sensing electrode, which causes an electric current. The current is converted to a voltage through a detection resistance, which will be an output of the sensing circuit. This paper provides a framework to analyze the output signal waveform and shows that the waveform consists of two components. One component is caused by driving voltage and appears regardless of the existence of a slider. The other component corresponds to the movement of a slider, which appears only when a slider is moving over the sensing electrode. Therefore, the slider can be detected by monitoring the latter component. The two components generally overlap, which makes the detection of the latter component difficult in some cases. This paper proposes a method to decouple the two components by switching the detection resistance at an appropriate time. These methods are verified using a prototype actuator that has an electrode pitch of 0.6 mm. The actuator was driven with a set of pulse voltages with an amplitude of 1000 V. The experimental results show similar waveforms to the analytical results, verifying the proposed analytical framework. The performance of the sensing method as a proximity sensor was verified in the experiments, and it was confirmed that the slider can be detected when it approaches the sensing electrode within about 3 mm.

## 1. Introduction

In recent years, electrostatic force has attracted much attention in the fields of robotics and human–computer interaction. One reason is that, unlike magnetic force, electrostatic force can be applied to a wide variety of materials, including dielectrics such as glass, as well as conductors. Another reason is that actuators utilizing electrostatic force can be made thin and transparent [1,2]. Utilizing such characteristics, various different applications have been proposed. In human–computer interaction, for example, many studies have tried to render tactile feeling on touch panel surfaces using the electrostatic attraction force that occurs between a human finger (or sometimes a pad operated by a finger) and the touch panel [3,4,5], which is known as electro-vibration. In the field of robotics, many studies have utilized electrostatic force for adhesion to walls, in the form of electro-adhesion devices [6,7,8,9]. In other robotic applications, electrostatic actuators show promise as powerful and flexible artificial muscles for robots [10,11,12,13,14].

The electrostatic actuators or devices used in these application areas have sizes ranging from millimeters to centimeters, unlike many other micro-scale electrostatic actuators in the MEMS field [15,16,17]. Electrostatic film actuators are some of these macro- or meso-scale electrostatic actuators. An electrostatic film actuator is realized using dielectric films, or sheets, that contain multi-phase electrodes. Various different configurations and driving principles have been proposed so far, which include charge induction [18], synchronous (variable capacitance) [10], capacitive voltage induction [19], and LC-resonant voltage induction [20,21]. Among these, the synchronous type showed superior output performance, such as thrust force in the order of a hundred newtons in a linear configuration [10,14]. Compared to other conventional types of electrostatic actuators, such as corona motors [22,23], liquid motors [24], and electret motors [25,26], the output performance of the synchronous film motors is found to be outstanding, due to their simple and light-weight structure realized by the use of thin sheets, which shows the advantage of utilizing films in electrostatic actuators.

This study addresses the *charge-induction type* electrostatic film actuator [18], which is another type of electrostatic film actuator. Although less powerful than the synchronous variant, the charge-induction type also has some unique characteristics coming from the use of films. In the charge-induction type, a slider is a sheet of dielectrics with no electrode structure. Since no electrode structure is required in a slider, some dielectric sheets, such as paper sheets and plastic sheets, can be directly utilized as a slider for this actuator, although there is a limitation in terms of surface resistivity. Therefore, the actuator can be utilized as a sheet feeder [27]. Using such a capability, unique applications have been proposed in the field of human interaction, which include desktop automation [2,28] and moving posters [29]. Although the desktop automation, in which documents and/or lightweight objects on a desktop will be automatically arranged, is still in the concept stage, the moving posters have actually been used for advertising purposes. In the moving poster application, a picture is printed on the stator and the slider of a large-size electrostatic actuator, which measures, for example, about 1 m in length. By actuating the slider sheet, a part of the picture can physically move. Although a similar effect can be realized using a large visual display in the form of digital moving images, the realism and the wonder of the moving poster attract people’s attention and make it highly effective in advertising.

Another unique characteristic of the film actuator is optical transparency. By utilizing transparent plastic sheets for the actuator material, the whole or a part of the actuators can be made optically transparent [1,2,30]. Together with the thin form factor, such a transparent actuator can be easily integrated with a visual display, comprising a vision–motion integrated display. Some prototypes have been demonstrated so far, in which a transparent actuator covers the surface of an LCD monitor to move a sheet or a lightweight object on the monitor in accordance with the computer graphics [2,31,32]. Such vision–motion integrated displays have been developed using several actuation principles, and the one using the induction principle [31,33,34] was found to be effective for interactive applications [31].

Because the charge-induction type is an asynchronous induction actuator, it is difficult to accurately grasp the position of the slider, which is an issue in the above-mentioned applications. In interactive applications, such as moving posters, the demand for precise displacement sensing is much lower than in industrial applications. However, for effective interaction, it is still important to detect a slider location with a relatively rough resolution. Proximity sensors would be a typical solution for such a purpose. However, the use of typical proximity sensors can ruin the very thin form factor of the actuator. Another popular solution would be using external cameras to detect the slider location. However, the use of such an external apparatus is not suitable in some applications where the thin and compact structure of the actuator is favored. In such situations, embedding a self-sensing function in the actuator is appreciated.

In electromagnetic motors, self-sensing for slider/rotor position has been widely studied [35,36,37]. Since there is a kind of duality between electromagnetic and electrostatic actuators, a similar self-sensing concept can be applied to electrostatic actuators. Various types of self-sensing, or built-in sensing, have been studied for electrostatic actuators, ranging from micro ones [38,39,40,41,42] to large-scale ones [43,44,45,46,47,48] including film actuators. Table 1 summarizes built-in sensing for film actuators. Although these film actuators have similar appearances, their operation principles and structural components are different. Therefore, a built-in sensing principle for one type cannot be simply transferred to another type. In particular, the charge-induction type is unique in that it utilizes a dielectric slider. Other electrostatic film actuators, like many other electrostatic actuators in general, utilize electrodes in their sliders, which allow the measurement of the capacitance between the stator and the slider. However, that is not always possible in the charge-induction type, since the slider has no electrode.

Even with the charge-induction type, built-in sensing through capacitance measurement is possible if the surface resistance of the slider is relatively small. In [44], for example, the capacitance between a slightly conductive surface of a dielectric slider and stator electrodes was measured. This was achieved by superposing a high-frequency sensing signal onto the low-frequency and high-voltage actuation signals by using transformers. The superposed signals are then applied to the stator electrodes and the high-frequency current runs through the surface of the slider, which facilitates the capacitance measurement. However, for this principle to work, the high-frequency current must flow in the slider, which requires the slider to have a relatively low surface resistance.

On the other hand, in the above-mentioned applications, relatively high surface resistance is often utilized for robust actuation. In charge-induction type actuators, charges are induced on the surface of a slider, from which electrostatic force is generated. The surface resistance of the slider affects the charge-induction speed; lower resistance realizes faster induction speed and vice versa. If the induction speed is faster, the induced charges can be more easily dissipated, resulting in unstable actuator behavior. On the other hand, if the induction speed is slower, the induced charges will be kept in a stable manner for a longer time, which realizes more stable actuator behavior. However, if the charges hardly move on the slider surface, slider detection is not possible with the capacitance measurement. Therefore, the existing built-in sensing cannot be applied to this case, and alternative sensing methods are needed.

In this paper, a method to detect a slider with a slower charge-induction speed is discussed. This method assumes that the charge induction is relatively slow, such as a charge-induction time of tens or hundreds of milliseconds, or even more. This means that the slider has a relatively high surface resistance. In such a case, the charges on the slider can be considered fixed on its surface during motor operation. Therefore, by utilizing the electrostatic induction caused by the fixed charges, the existence of a slider can be detected. This paper proposes an analytical framework to analyze such electrostatic induction to show that slider detection really is possible. This paper also proposes a way to enhance the detection, which is realized through resistance switching in the sensing circuit.

The rest of this paper is structured as follows. In Section 2, the principle of the charge-induction type electrostatic actuator will be explained in terms of its linear configuration. Section 3 introduces the concept of self-sensing and proposes the analytical framework. In the proposed framework, the electrostatic induction is analyzed using a capacitance network model. In Section 4, analytical results using the proposed framework are shown. The results show that the existence of a slider can be detected through electrostatic induction. The section also discusses a necessary modification to the analytical model, such that the analysis can better explain the experimental results. Section 5 discusses an improvement of the detection using resistance switching. In Section 6, the detection methods discussed in the previous sections are demonstrated using a prototype actuator. The results confirm that output signals that are considerably similar to the numerical analyses can be obtained in the real actuator, verifying the feasibility of the detection method. Finally, Section 7 provides conclusions.

## 2. Charge-Induction Electrostatic Actuator

**Figure 1:**
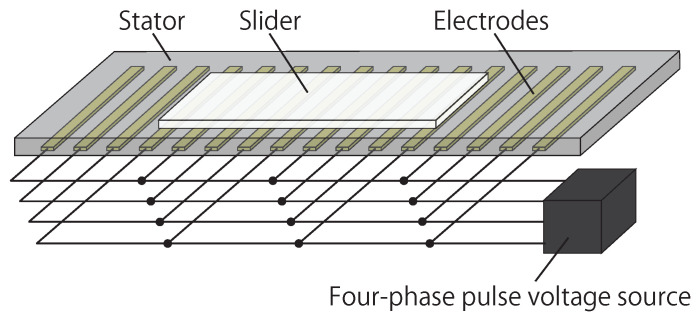
Structure of charge-induction electrostatic linear actuator with a four-phase stator.

The basic structure of the charge-induction electrostatic actuators is shown in Figure 1, using a linear configuration. The actuator utilizes multi-phase parallel electrodes embedded in its stator. The parallel electrodes are arranged with a regular pitch. The pitch depends on fabrication, and ranges between ca. 100 μm and 1 mm. In this particular work, we focus on four-phase parallel electrodes, but the concept proposed in this work can be applied to other variants, which include three-phase and six-phase electrode structures. A stator can be fabricated using a printed circuit board (PCB) manufacturing process, or by printing electrodes on sheet materials using printers. Regardless of the fabrication processes, the surface of the electrodes is covered with an insulating layer.

On the stator substrate, a slider sheet is arranged, which is to be actuated by electrostatic force. A slider is a dielectric sheet that does not have electrodes, but has a slight conductivity on its surface, such as, for example, $10^{13}\Omega$ of surface resistivity. Some dielectric sheets naturally have such surface resistivity, but in most cases, the surface of a slider is treated to adjust surface resistivity.

**Figure 2:**
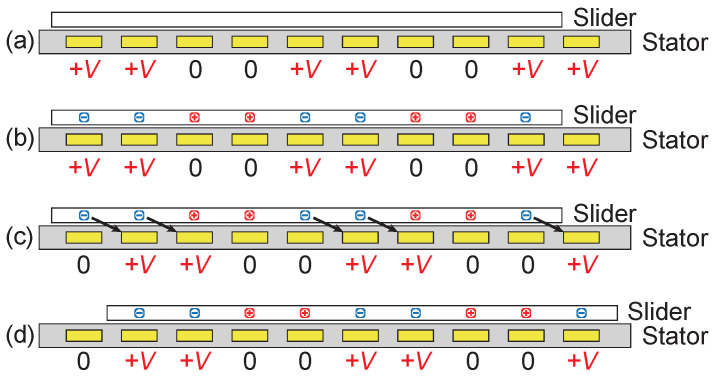
Driving principle of charge-induction electrostatic actuators using a mono-polar high voltage. The voltage, *V*, is typically around 1 kV. (**a**) Voltages are applied to the stator electrodes; (**b**) charges are induced on the slider surface; (**c**) voltages on the stator electrode are shifted; (**d**) the slider moves about one electrode pitch.

The driving operation for the four-phase actuator is shown in Figure 2. The actuator can be operated using a set of two DC voltages, 0 and a positive high voltage, *V*. First, the voltages are applied to the four-phase stator electrodes, as in Figure 2a. This voltage pattern is denoted as [V,V,0,0]. The voltage application creates a spatially cyclic voltage pattern with a period of four electrode pitches, as shown in the figure. The stator voltage pattern causes surface current to flow on the slider surface. The surface current creates a charge pattern as in Figure 2b, which is a mirrored pattern of the voltage pattern in the stator. Here we assume that this charge-induction process takes a few tens or hundreds of milliseconds or more, due to the surface resistivity.

Then, the stator voltages are shifted to [0,V,V,0], which shifts the location of the spatial voltage pattern by one pitch, as in Figure 2c. The shift induces an electrostatic force on the charges on the slider. Since the charge induction takes a few hundred milliseconds, in other words, the charge motions are considerably slow, we can assume that the charges are fixed on the surface for a short period after the voltage shift. As a result, the electrostatic force moves the whole slider, not just charges, as shown in Figure 2d. As will be demonstrated later in the experimental section, this slider movement takes, for example, about ten milliseconds, which is shorter than the charge-induction time.

Repeating the above process will move the slider continuously in a step-wise trajectory. Typically, the process is repeated at a rate of several to several dozen times per second. In an ideal condition, the slider moves one electrode pitch at each voltage shift. However, in reality, the charge pattern may slip on the slider surface during each step movement, or friction may impede the slider motion. Such a slip and/or friction will change the step length, which makes the prediction of the slider location difficult in an open-loop control.

## 3. Slider Detection Using Stator Induction Current

### 3.1. Basic Concept

**Figure 3:**
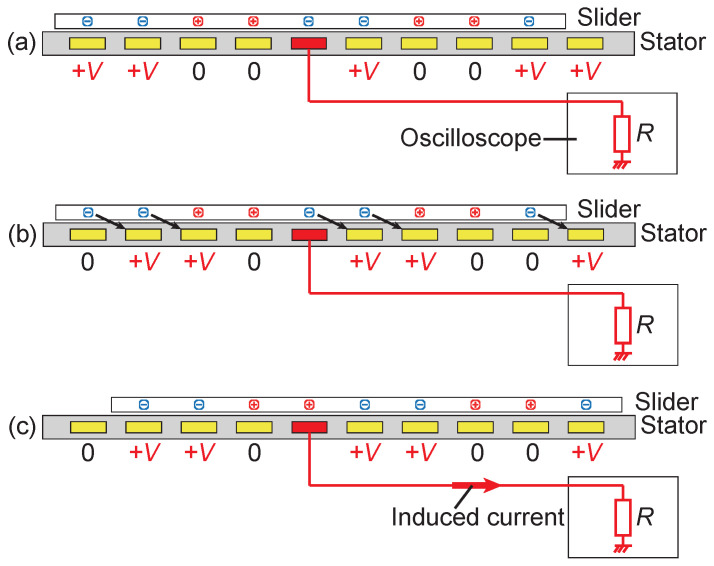
The slider detection method analyzed in this work. The red electrode is utilized as a sensing electrode. The induced current is monitored by an oscilloscope to detect the slider motion over the sensing electrode. (**a**) A charged slider is on the stator surface; (**b**) the stator voltages are shifted; (**c**) the slider moves and the charges on the slider induce current in the sensing electrode.

The method for detecting a slider assumes that one of the stator electrodes is disconnected from the other electrodes and dedicated to sensing, as in Figure 3. When the slider with an induced charge pattern moves over the sensing electrode, as shown in Figure 3b,c, electric charges are induced on the sensing electrode, which causes an electric current. By detecting this current using a resistance, which can be an input resistance of an oscilloscope, as in the figure, the existence of the slider can be detected. As described in the later results, the measured voltage is typically much less than 100 V for the actuator’s driving voltage of around 1 kV. If we use 10 MΩ for example, the power consumed at the resistance is 1 mW, which means typical resistors with 1/4 W can be utilized for this purpose. It should also be noted that the measurement circuit has some capacitance. The capacitance is ignored in the following analysis for simplicity and will be further discussed in Section 6.5.

### 3.2. Analytical Model

**Figure 4:**
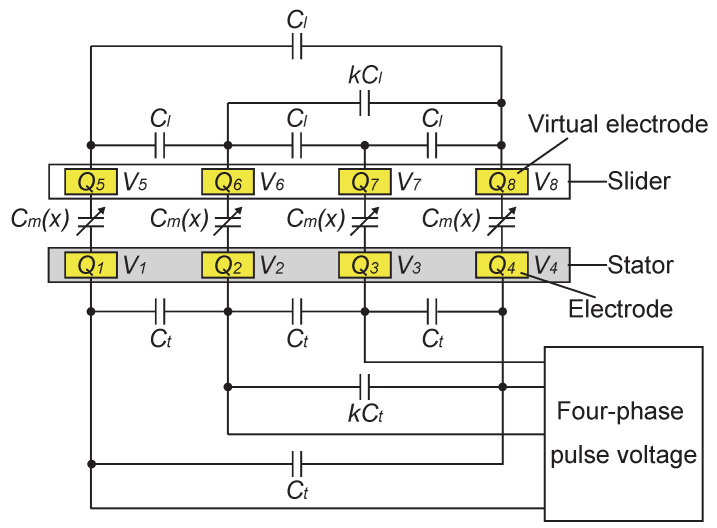
Analysis model of the electrostatic actuator. The model represents one cycle (four electrode pitches) of the structural repetition of the actuator. Although capacitance exists between any two electrodes, some of them are omitted to avoid crowding the figure.

The above concept is analyzed using a capacitance network model, which is expanded from the model for a three-phase synchronous motor [49]. The model is used for analyzing the induction current on the sensing electrode during the slider movement. Here, we assume that the charge pattern on the slider remains constant during the movements of the slider. The assumed charge pattern is the mirrored pattern of the stator voltage. As containers for these charge patterns, we arrange virtual electrodes in the slider. The resulting model is graphically shown in Figure 4. The model represents one cycle (four electrode pitches) of the structural repetition of the actuator. In the figure, some capacitors are omitted to avoid crowding the figure, but all the electrodes are connected by capacitors. The capacitances between the stator and the slider vary depending on the slider displacement, whereas the capacitances among stator electrodes, as well as among slider virtual electrodes, are constant regardless of the slider displacement.

Now, we assign numbers 1 through 4 to the stator and 5 through 8 to the slider electrodes and let $Q_{1}$ to $Q_{8}$ and $V_{1}$ to $V_{8}$ represent the charges and voltages of the electrodes. Then, the relationship between a charge vector $Q={\left(Q_{1},\ldots ,Q_{8}\right)}^{t}$ and a voltage vector $V={\left(V_{1},\ldots ,V_{8}\right)}^{t}$ is expressed as

(1) Q=C(x)V

where *x* is a normalized slider displacement and superscript *t* for a vector (or a matrix) represents transpose. In the normalized displacement, four electrode pitches, which are the periodic cycle, are normalized to 2π. The matrix C(x) is the capacitance matrix representing all the capacitances among the eight electrodes. It is a symmetric 8-by-8 matrix, whose diagonal element, $C_{ii}$, represents the self-capacitance of the *i*th electrode, and an off-diagonal element, $C_{ij}$, represents the capacitance between the *i*th and *j*th electrode by its absolute value. It should be noted that off-diagonal elements always take negative values due to the nature of the capacitance matrix. Considering the structure of the motor, the matrix can be decomposed as

(2) $$\begin{array}{cc} C(x)= & \left(\begin{array}{cc} T & M(x)\\ M^{t}\left(x\right) & L \end{array}\right) \end{array}$$

The 4-by-4 partial matrix T represents the capacitances among the stator electrodes by its off-diagonal elements, whereas L represents those among the slider ones. The 4-by-4 partial matrix M(x) represents the capacitances between the stator and the slider electrodes.

From the geometrical arrangement of the electrodes, these partial matrices can be written as

(3) $$\begin{array}{cc} M(x)= & \left(\begin{array}{cccc} C_{m}\left(x\right) & C_{m}\left(x+\frac{\pi }{2}\right) & C_{m}\left(x+\pi \right) & C_{m}\left(x+\frac{3\pi }{2}\right)\\ C_{m}\left(x+\frac{3\pi }{2}\right) & C_{m}\left(x\right) & C_{m}\left(x+\frac{\pi }{2}\right) & C_{m}\left(x+\pi \right)\\ C_{m}\left(x+\pi \right) & C_{m}\left(x+\frac{3\pi }{2}\right) & C_{m}\left(x\right) & C_{m}\left(x+\frac{\pi }{2}\right)\\ C_{m}\left(x+\frac{\pi }{2}\right) & C_{m}\left(x+\pi \right) & C_{m}\left(x+\frac{3\pi }{2}\right) & C_{m}\left(x\right) \end{array}\right) \end{array}$$

(4) $$\begin{array}{cc} T= & (\begin{array}{cc} 4C_{0}-2C_{a}+\left(k+2\right)C_{t} & -C_{t}\\ -C_{t} & 4C_{0}-2C_{a}+\left(k+2\right)C_{t}\\ -kC_{t} & -C_{t}\\ -C_{t} & -kC_{t} \end{array}\\ \; & \;\;\;\begin{array}{cc} -kC_{t} & -C_{t}\\ -C_{t} & -kC_{t}\\ 4C_{0}-2C_{a}+\left(k+2\right)C_{t} & -C_{t}\\ -C_{t} & 4C_{0}-2C_{a}+\left(k+2\right)C_{t} \end{array}) \end{array}$$

(5) $$\begin{array}{cc} L= & (\begin{array}{cc} 4C_{0}-2C_{a}+\left(k+2\right)C_{l} & -C_{l}\\ -C_{l} & 4C_{0}-2C_{a}+\left(k+2\right)C_{l}\\ -kC_{l} & -C_{l}\\ -C_{l} & -kC_{l} \end{array}\\ \; & \;\;\;\begin{array}{cc} -kC_{l} & -C_{l}\\ -C_{l} & -kC_{l}\\ 4C_{0}-2C_{a}+\left(k+2\right)C_{l} & -C_{l}\\ -C_{l} & 4C_{0}-2C_{a}+\left(k+2\right)C_{l} \end{array}) \end{array}$$

In M(x), which is an off-diagonal part of C(x), any element in the partial matrix represents the capacitance between one stator electrode and one slider electrode by its absolute value, which is expressed as

(6) $$C_{m}\left(x\right)=\left\{\begin{array}{cc} -C_{0}-C_{a}\cos2x & \left(x\leq \frac{\pi }{2},\frac{3\pi }{2}\leq x\right)\\ -C_{0}+C_{a} & \left(\frac{\pi }{2}<x<\frac{3\pi }{2}\right) \end{array}\right.$$

Any pair of a stator and a slider electrode exhibits the same relationship but with a shift in the location. Therefore, all the elements in M(x) are expressed by shifting the variable *x* of the same function. Here, it should be remembered that the displacement *x* is the normalized displacement where the distance of four pitches, which is the periodic cycle, is normalized to 2π.

In T, an off-diagonal element represents the capacitance between two stator electrodes by its absolute value. The capacitance between any two adjacent electrodes was assumed to be $C_{t}$ and that between non-adjacent electrodes to be $kC_{t}$, with 0<k<1. The same explanation applies to L, which is the matrix for the slider electrodes.

To define the diagonal elements of T and L, we assumed that the actuator is a closed system in terms of the electric field. This assumption forces a sum of any row or column of the total capacitance matrix C(x) to be zero. Therefore, the diagonal elements of T and L were set to satisfy such a condition. For example, for the first row of C(x), the sum of the off-diagonal elements of the first row of T is $-2C_{t}-kC_{t}$. The sum of the first row of M(x) is $-4C_{0}+2C_{a}$. Therefore, the first diagonal element of T was set as the negative value of the sum of the above two, which is $4C_{0}-2C_{a}+\left(k+2\right)C_{t}$.

### 3.3. Procedure of the Analysis

As explained in the driving principle, the actuator is driven in the following steps: (1) charging the slider by applying a set of constant voltages to the stator electrodes, (2) shifting the stator voltages, and (3) the slider being moved step-wise. First, we calculate how many charges are induced in the virtual electrodes in step (1).

Since we assume that the induced charges are in a steady state and constant, we ignore the existence of the sensing electrode in calculating the steady-state charges. The set of DC voltages is applied to the four stator electrodes until the charge induction reaches a steady state. In the steady state, the slider virtual electrodes should have the average voltage of the four stator electrodes, which is $V_{0}/2$, with $V_{0}$ being the high voltage used for the driving. The resulting voltage vector is denoted as $V^{init}$, which is expressed as

(7) $$V^{init}={\left(V_{0},V_{0},0,0,V_{0}/2,V_{0}/2,V_{0}/2,V_{0}/2\right)}^{t}$$

for the stator voltage pattern of $\left[V_{0},V_{0},0,0\right]$. Then, the charges on all the electrodes, $Q^{init}$, can be calculated as

(8) $$Q^{init}=C\left(0\right)V^{init}$$

After the charges are determined, the sensing electrode is introduced into the analysis. This work assumes that electrode 2 is used as the sensing electrode. Since the charges on the slider are known as $Q_{5}^{init}$ to $Q_{8}^{init}$ and the voltage at the sensing electrode should be 0 V, the system should now fulfill the following relation

(9) $$\begin{array}{c} {\left(Q_{1}^{sen},Q_{2}^{sen},Q_{3}^{sen},Q_{4}^{sen},Q_{5}^{init},Q_{6}^{init},Q_{7}^{init},Q_{8}^{init}\right)}^{t}\\ =C\left(0\right){\left(V_{0},0,0,0,V_{5}^{sen},V_{6}^{sen},V_{7}^{sen},V_{8}^{sen}\right)}^{t} \end{array}$$

Here, it should be noted that the charges on the stator electrodes ($Q_{1}^{sen}$ to $Q_{4}^{sen}$), as well as the voltages of the slider virtual electrodes ($V_{5}^{sen}$ to $V_{8}^{sen}$), are different from $Q^{init}$ and $V^{init}$ and, thus, unknown, since the voltage on electrode 2 is changed from the previous step. Therefore, we obtain $Q_{1}^{sen}$ to $Q_{4}^{sen}$ and $V_{5}^{sen}$ to $V_{8}^{sen}$ by solving the above equation.

In the next step, the voltages on the stator driving electrodes are shifted to $\left[0,V_{0},V_{0},0\right]$. Here, it should be noted that electrode 2 in the model is a sensing electrode, and the voltage of $V_{0}$ is not applied; the voltage on the sensing electrode is determined passively, which is denoted as $V_{2}\left(t\right)$ as a function of time *t*. The voltages on the slider virtual electrodes will also change with the voltage shift, and are denoted as $V_{5}\left(t\right)$ to $V_{8}\left(t\right)$. As we assumed that the charges on the slider do not change during a slider movement, the charges on the slider are kept as $Q_{5}^{init}$ to $Q_{8}^{init}$. The charges on electrode 2 at the moment right after the voltage shift should be the same as before the voltage shift, and, thus, are $Q_{2}^{init}$$\left(=Q_{2}\left(0\right)\right)$. Using these quantities, the following equation can be written for the moment right after the switching, or t=0.

(10) $$\begin{array}{c} {\left(Q_{1}\left(0\right),Q_{2}^{init},Q_{3}\left(0\right),Q_{4}\left(0\right),Q_{5}^{init},Q_{6}^{init},Q_{7}^{init},Q_{8}^{init}\right)}^{t}\\ =C\left(0\right){\left(0,V_{2}\left(0\right),V_{0},0,V_{5}\left(0\right),V_{6}\left(0\right),V_{7}\left(0\right),V_{8}\left(0\right)\right)}^{t} \end{array}$$

At this step, the slider is not yet moved and, therefore, the displacement *x* is assumed to be zero. By solving this equation for $Q_{1}\left(0\right)$, $Q_{3}\left(0\right)$, $Q_{4}\left(0\right)$, $V_{2}\left(0\right)$, and $V_{5}\left(0\right)$ through $V_{8}\left(0\right)$, we obtain all the quantities at t=0.

Finally, we calculate how the voltages and charges will change during a slider movement. For this purpose, a slider movement is defined as a function of time, x(t), and the following set of equations is solved with the initial condition obtained for t=0.

(11) $$\left\{\begin{array}{c} \frac{d}{dt}Q_{2}\left(t\right)=-\frac{V_{2}\left(t\right)}{R}\left(=i_{2}\left(t\right)\right)\\ Q^{m}\left(t\right)=C\left(x(t)\right)V^{m}\left(t\right) \end{array}\right.$$

where

(12) $$\begin{array}{ccc} Q^{m}\left(t\right) & = & {\left(Q_{1}\left(t\right),Q_{2}\left(t\right),Q_{3}\left(t\right),Q_{4}\left(t\right),Q_{5}^{init},Q_{6}^{init},Q_{7}^{init},Q_{8}^{init}\right)}^{t} \end{array}$$

(13) $$\begin{array}{ccc} V^{m}\left(t\right) & = & {\left(0,V_{2}\left(t\right),V_{0},0,V_{5}\left(t\right),V_{6}\left(t\right),V_{7}\left(t\right),V_{8}\left(t\right)\right)}^{t} \end{array}$$

The first equation in (11) describes the current on electrode 2, $i_{2}\left(t\right)$, and *R* represents the resistance to measure the current. Among the obtained solutions, $V_{2}\left(t\right)=Ri_{2}\left(t\right)$ is the output of the sensing circuit.

## 4. Analytical Results

### 4.1. Behavior of the Proposed Model

The above equations were numerically solved for the following condition using Mathematica (Version 12.2, Wolfram Research, Champaign, IL, USA).

(14) $$\left\{\;\begin{array}{cc} k & =0.01\\ C_{0} & =6\;\mathrm{pF}\\ C_{a} & =6\;\mathrm{pF}\\ C_{l} & =50\;\mathrm{pF}\\ C_{t} & =50\;\mathrm{pF}\\ V_{0} & =1000\;V\\ R & =10\;M\Omega \end{array}\right.$$

The displacement of the slider for one step was defined as

(15) $$x\left(t\right)=\frac{\pi }{2\left(\exp\left(-1500(t-0.003)\right)+1\right)}$$

For comparison, we also calculated $V_{2}\left(t\right)$ for a case when a slider does not exist near the sensing electrode. Such a case can be represented by setting the slider charges, $Q_{5}^{init}$ to $Q_{8}^{init}$, and x(t) to be zero.

**Figure 5:**
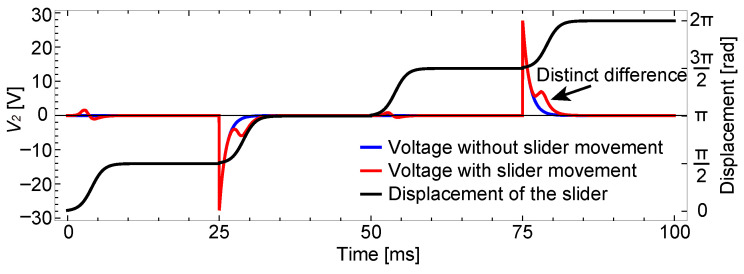
Analytical results for four consecutive motion steps. The slider moves one electrode pitch, which is π/2 in the normalized displacement, at every voltage shift. The red line shows the result when a slider is moving over the sensing electrode, and the blue line shows that without a slider. From the difference between the two output waveforms, the existence of the slider can be detected.

**Figure 6:**
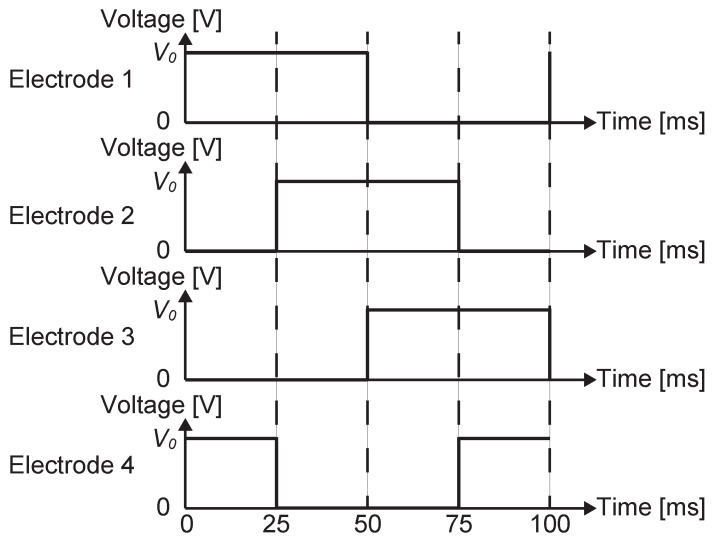
The voltage patterns applied to stator electrodes. Each pattern is a pulse wave with a 50% duty ratio. Voltage shift occurs every 25 ms in two of the four patterns. The voltage for “Electrode 2” is applied to the electrodes belonging to the second phase, except the sensing electrode.

The results are shown in Figure 5. The analysis assumed that the voltage pattern shown in Figure 6 is applied to the stator electrodes. Since the procedure described in the previous section is only for a single voltage shift, it was repeated by changing the voltage conditions to obtain this result.

The red and blue lines in Figure 5 represent the calculated output, $V_{2}\left(t\right)$, for two different cases: with and without a slider. As the current flows in the sensing electrode even without the slider, similar waveforms appear in the two cases. However, there are distinct differences in the waveforms at around 30 and 80 ms. Therefore, by identifying these two lines based on the difference, the existence of the slider on the sensing electrode can be detected.

The signal difference between the two cases appears four times in one cycle of the voltage pattern. However, the differences in the first and the third voltage shifts, at around 3 and 53 ms, are not as significant as those for the second and fourth voltage shifts. Considering the existence of the noise, as well as the disturbed waveform discussed in the next subsection, the first and the third differences might be difficult to detect, and, thus, the second and the fourth differences would be suitable for sensing. It should also be noted that if a slider exists on the sensing electrode but does not move, possibly due to some disturbance, the output will be the same as the case without a slider. Therefore, the detection method will detect that “a slider exists and is moving” over the sensing electrode.

### 4.2. Modified Model

As shown later in the experimental section, the above result does not perfectly describe the experimental results. This is because, in a real actuator, the capacitances among stator electrodes are not completely balanced. To better explain the experimental results, an additional analysis was performed using a modified capacitance matrix. In the modified matrix, the partial matrix ***T*** is changed to $\hat{T}$.

(16) $$\hat{T}=\left(\begin{array}{cccc} C_{t0} & -bC_{t} & -kC_{t} & -C_{t}\\ -bC_{t} & C_{t0} & -C_{t} & -kC_{t}\\ -kC_{t} & -C_{t} & C_{t0} & -bC_{t}\\ -C_{t} & -kC_{t} & -bC_{t} & C_{t0} \end{array}\right)$$

where $C_{t0}=4C_{0}-2C_{a}+\left(k+b+1\right)C_{t}$. The parameter *b* represents the imbalance.

**Figure 7:**
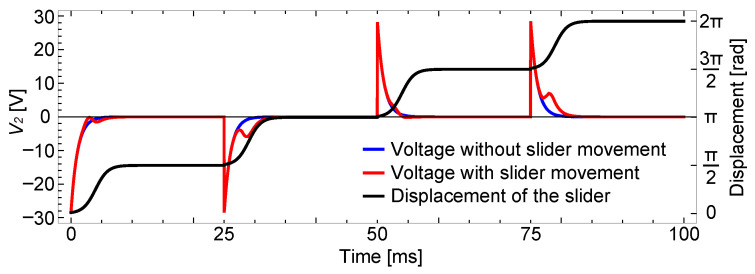
Analytical results with the capacitance imbalance. The parameter *b* for describing the imbalance was set to 0.94.

The results when *b* is set to 0.94 are shown in Figure 7.

As shown in this plot, when there is a capacitance imbalance, the voltage on the sensing electrode shows large spikes for all the voltage shifts, which is different from the previous result and better explains the experimental results described later. The spikes for the first and the third voltage shifts almost hide the subtle difference between the two cases, with and without a slider.

## 5. Resistance Switching for Robust Detection

### 5.1. Relationship between the Resistance of Sensing Circuit and Output Signal

The above results clearly showed differences in output signals with and without a slider. However, the output signal can be affected by various factors, such as the slider motion profile or the gap fluctuation between the stator and the slider. Because of such susceptibility, detecting the difference may not be always easy. For robust detection, the difference in the output signals should be far more distinct. This section discusses the characteristics of the output signal in order to enhance the difference. In the following, the fourth voltage shift is focused on.

**Figure 8:**
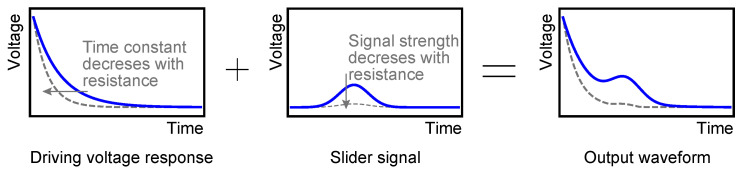
The output waveform consists of two components, the driving voltage response and the slider signal. The driving voltage response appears right after the voltage shift, whereas the slider signal reaches its peak when the slider speed reaches the maximum. Both components depend on the resistance of the sensing circuit. For example, when the resistance is decreased, the waveforms will change to gray dashed lines.

The above results showed that even when there is no slider, a similar waveform appears in the output. This is because the shifting of the stator voltages induces a current in the sensing electrode. The induced current gradually decays, with a time constant defined by the capacitance of the electrodes and the resistance of the sensing circuit. This decaying waveform is hereafter referred to as *driving voltage response*. On the other hand, when the slider moves over the sensing electrode, an additional current flows in accordance with the slider motion, which makes the difference in the output waveforms. Hereafter, this additional signal is referred to as *slider signal*. The final output is the sum of the driving voltage response and the slider signal, as in Figure 8, and the purpose of the sensing circuit is to identify the small slider signal in the large driving voltage response.

To detect the slider signal under the existence of the driving voltage response in a robust manner, this work tries to separate them in time. The separation would be realized by letting the driving voltage response decay much faster. Faster decay can be realized by setting a smaller resistance in the sensing circuit. If the resistance is set smaller, such that the driving voltage response decays before the slider motion is initiated, the two waveform components will be separated.

However, reducing the resistance has a side effect, which is a decrease in the slider signal. Since the output voltage is a product of the current and the resistance, a smaller resistance can only produce a smaller output signal, which might be hidden in the noise. This means that there is a trade-off between the decay of the driving voltage response and the strength of the slider signal.

### 5.2. Resistance Switching

The above-mentioned trade-off can be resolved by switching the resistance at a proper moment. First, the resistance is set small, such that the driving voltage response decays in a short time. Then, before the slider starts to move, the resistance is switched to a larger value, such that a large slider signal can be obtained.

The response when the resistance is switched can be analyzed using the method in Section 3.3 with a slight modification. First, the differential equation in (11) is solved using the small resistance until the pre-defined moment of resistance switch. Then, the charges $Q_{1}$ to $Q_{4}$ and $V_{5}$ to $V_{8}$ at the moment of the resistance switch are recorded and used as the initial condition for the next step. In the next step, the same differential equation is solved for the time after the resistance switch, using the large resistance value and the initial condition obtained in the previous step.

**Figure 9:**
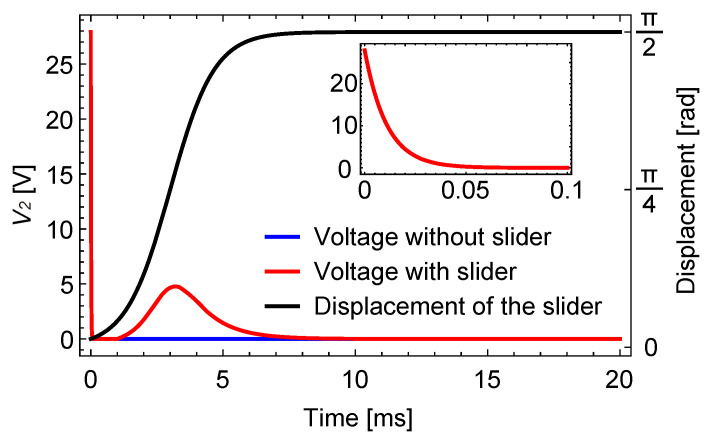
Analytical result for the resistance switching. The resistance *R* was switched from 100 kΩ to 10 MΩ at 1 ms. The result corresponds to the fourth voltage shift (at 75 ms) in Figure 6. The inset plot magnifies the response at the beginning.

The results of the analysis using the same parameters in Figure 5, except for the resistance value, are shown in Figure 9 for one voltage shift. Here, the resistance was switched from 100 kΩ to 10 MΩ at 1 ms after the voltage shift.

The time constant for the decay of driving voltage response was 4.9 μs, and the response almost completely decayed before the resistance switch. On the other hand, the slider motion was not yet significant at the moment of the resistance switch. The slider speed increased after that and the slider signal grew significantly due to the large resistance.

As the slider signal is separated from the driving voltage response, the existence of the slider can be easily detected by monitoring the sensing signal after the resistance switch. For example, setting a threshold of around 2 to 3 V will quite easily distinguish the two cases. This result indicates that switching the resistance value is effective for separating the two waveform components, which should contribute to the robust detection of the slider signal in real situations.

## 6. Experiment

### 6.1. Experimental Setup

**Figure 10:**
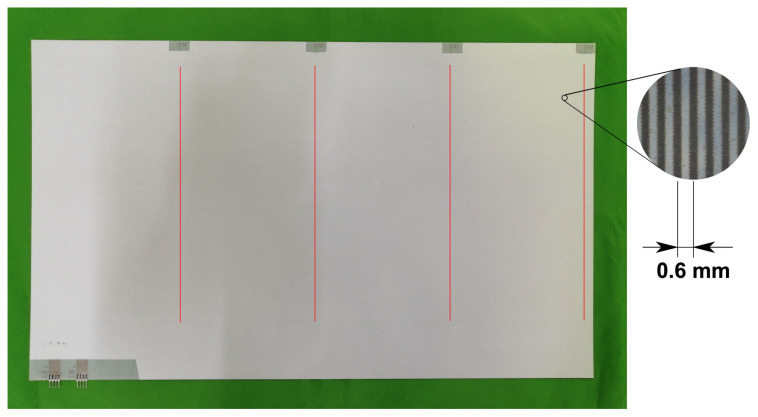
The stator of the electrostatic actuator used in the experiment. The white rectangle is the stator sheet. Although not visible due to the opaque insulation layer, the stator sheet contains four-phase parallel electrodes with an electrode pitch of 0.6 mm, which is shown in the enlarged picture on the right. Four sensing electrodes are arranged at the locations shown by red lines. The red lines were drawn in the photo for explanation purposes.

**Figure 11:**
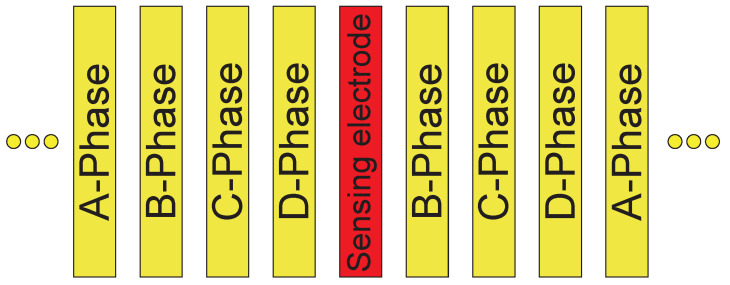
Arrangement of the stator electrodes. The stator electrodes are connected in 4 phases, such that the 4 phases appear repeatedly in the same order. The sensing electrode is electrically independent and is not connected to the 4-phase. The phase that was originally assigned to the sensing electrode is skipped.

A stator with a size of approximately 500 mm × 300 mm, shown in Figure 10, was used for the experiment. The stator had parallel electrodes with an electrode pitch of 0.6 mm. All the electrodes, except four independent electrodes, were connected in 4-phase, in the manner shown in Figure 11. The four independent electrodes, which were distributed in their locations, were intended for use as a sensing electrode. The following experiment utilized the third one from the left. The other independent electrodes were connected to the electric ground.

**Figure 12:**
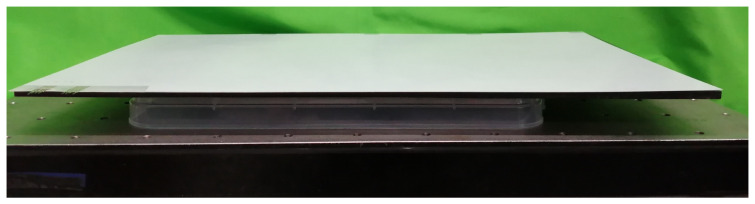
Stator arrangement. On an electrically-grounded metal plate, a white–translucent plastic box was placed to reduce capacitance coupling. Then, a black polyacetal plate and the stator sheet were placed on top.

The experiment was conducted on a metal surface plate, which was electrically grounded. To reduce the capacitance coupling between the surface plate and the stator, a plastic box with a height of 27 mm was arranged on the metal surface plate. As the stator sheet was flexible, it was attached to a rigid polyacetal plate and then placed on the plastic box, as shown in Figure 12.

**Figure 13:**
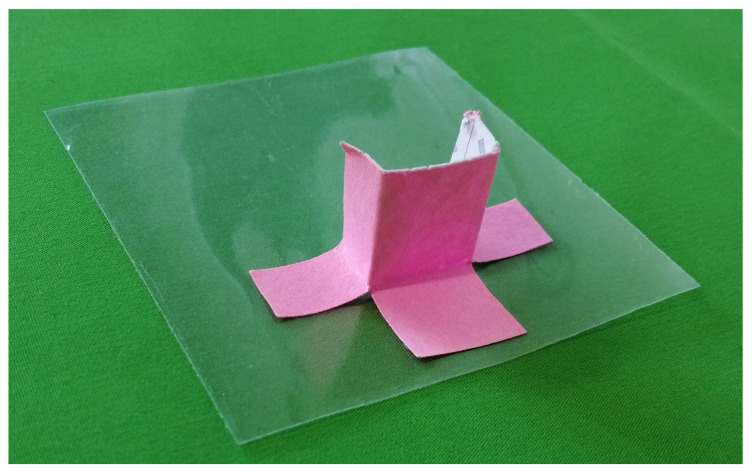
Slider with cardboard. The transparent sheet was the slider used in the work. The pink cardboard was attached using tape to facilitate displacement measurement using a laser displacement sensor.

A 70 mm × 70 mm dielectric sheet was used as the slider. As shown in Figure 13, a piece of cardboard was attached to the slider using double-sided tape to facilitate measurement using a laser displacement meter (OMRON, ZX1-LD100A81).

### 6.2. Measurement without Resistance Switch

First, the voltage of the sensing electrode during the slider movement was measured without the resistance switch. The sensing electrode was connected to an oscilloscope (Keysight, DSOX2004A) via a high-voltage probe (Tektronix, P5100) with an attenuation ratio of 100:1, which provides a nominal input impedance of 10 MΩ. For driving, the four-phase pulse signal shown in Figure 6 with an amplitude of 1 V was generated by a function generator (YOKOGAWA, AG1200) and was amplified 1000-fold using four high-voltage amplifiers (NF Corporation, HVA-4321). The resulting driving voltage, $V_{0}$, was 1 kV. The amplified voltage set was applied to the 4-phase stator electrodes.

**Figure 14:**
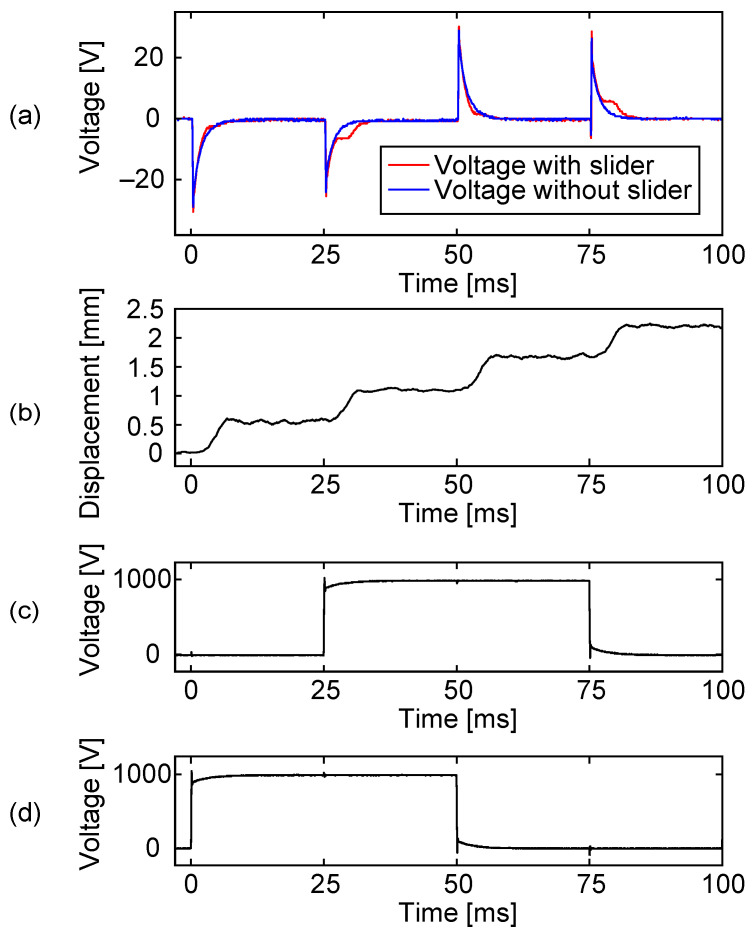
The voltage of the sensing electrode during slider motion when the resistance switch was not utilized. The sensing electrode was directly connected to an oscilloscope via a 100:1 passive probe. Plot (**a**) shows the sensing electrode voltage in red. For comparison, the sensing electrode voltage was measured without a slider (blue). Plot (**b**) shows slider motion. Ideally, the slider should move 0.6 mm at every voltage shift, but the actual step length was found to be slightly smaller for this particular case. Plots (**c**,**d**) are two voltages out of four applied to the stator electrodes.

The results are shown in Figure 14. In (a), the driving voltage responses appeared in both cases, with and without a slider, which are shown using red and blue lines, respectively. The response appeared at all the voltage switches, the same as in Figure 7, which indicates the existence of the capacitance imbalance. When there was a slider that moved as shown in (b), the slider signal appeared at around 30 and 80 ms. On the other hand, when there was no slider, no slider signal appeared and the two cases could be clearly distinguished.

**Figure 15:**
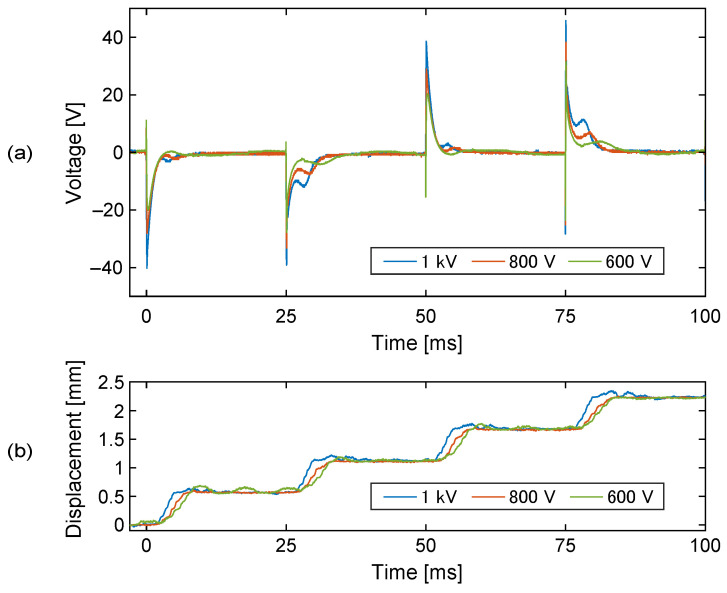
The voltages of the sensing electrode (**a**) and the slider displacement (**b**) when the actuator was operated by different driving voltage amplitudes, 600, 800, and 1000 V. As the driving voltage amplitude became smaller, the slider speed during the step movement became slower. As a result, the slider signal became weaker.

Figure 15 shows the voltage of the sensing electrode when the slider was driven using different voltage amplitudes. As the driving voltage was lowered, the step movement of the slider became slower. This affected the magnitude, as well as the timing, of the slider signal. Since the amplitude of the slider signal depends on the instantaneous speed of the slider, the slower step movement could produce a smaller slider signal. In a practical situation, even if the actuator is driven using a sufficiently high voltage, the slider speed could be occasionally reduced by some disturbances, which would also result in a weak slider signal. This indicates the need for the resistance switching.

### 6.3. Resistance Switching

**Figure 16:**
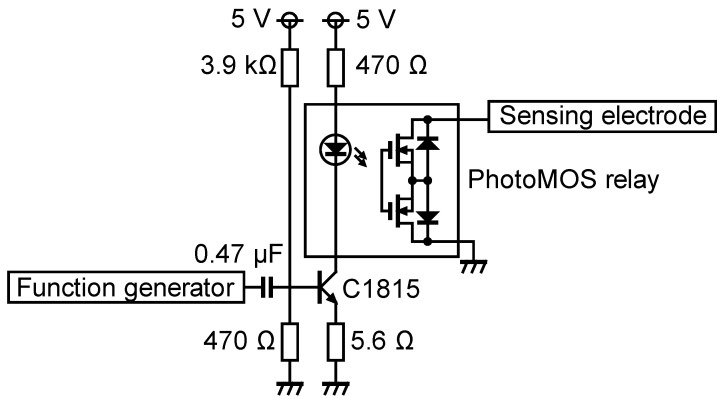
Circuit for switching the resistance of the sensing circuit. Panasonic AQV258 was used as the PhotoMOS relay, which has the rated peak current of 0.06 A. The maximum current from the sensing electrode may have exceeded this value in the experiment, although no operation problem was observed. Additional resistance may be added in series for reducing the current.

Next, an experiment with the resistance switching was conducted. Resistance switching was performed using the circuit shown in Figure 16.

In this circuit, a driving pulse waveform (the one for electrode 4 in Figure 6, but before 1000-fold amplification) from the function generator switched the transistor (2SC1815), utilizing the rising edges of the pulse waveform. The combination of the capacitance and the resistance connected to the base of the transistor kept the transistor on for about 0.2 ms. This means that 0.2 ms after the voltage shift on the stator electrodes, the resistance of the sensing circuit was changed.

The sensing electrode was connected to the resistance switching circuit, which was then connected to the oscilloscope via the 100:1 probe. When the transistor and the solid state relay (Panasonic PhotoMOS relay AQV258) were on, the sensing electrode was connected to the ground through the internal resistance of the relay, which was in the order of hundreds Ω. On the other hand, when the relay was off, the input resistance was that of the oscilloscope–probe combination.

**Figure 17:**
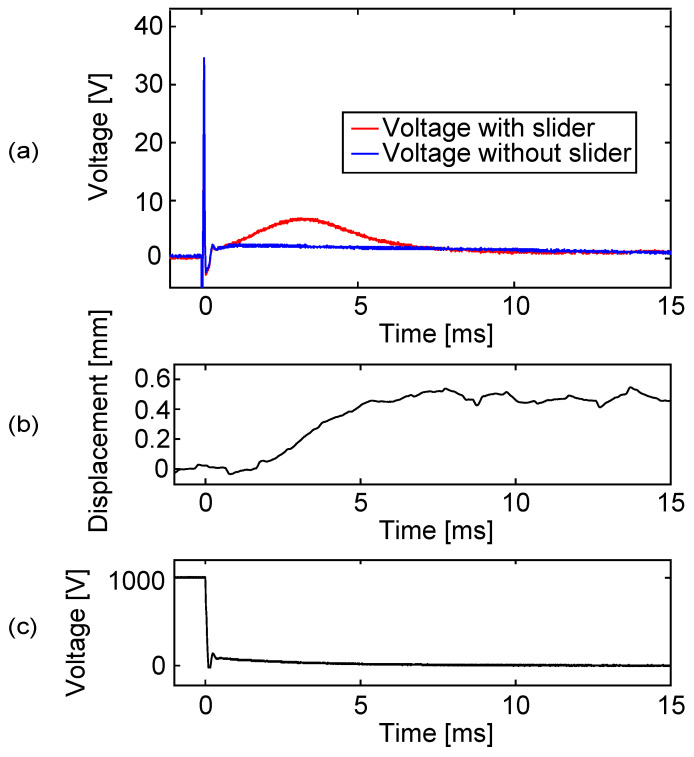
The voltage at the sensing electrode (**a**) when the resistance of the sensing circuit changed at 0.2 ms after the driving voltage shift. Red and blue lines compare the outputs for the two cases, with and without the slider. The plot for the slider displacement in (**b**) shows that the step length was about 0.5 mm for this particular case, which is smaller than one electrode pitch of 0.6 mm. The plot in (**c**) shows the voltage waveform for electrode 2.

Figure 17 shows the voltage change at the sensing electrode when the resistance was switched using this circuit. This result corresponds to the fourth voltage shift in Figure 14.

The plot shows the two cases, with and without a slider, using blue and red lines, respectively. In both cases, the driving voltage response that appeared at 0 s decayed almost immediately due to the low resistance. Then, the resistance was switched to the larger one, such that the sensing signal can be clearly observed. The slider movement shown in (b) was delayed after the voltage switch for about 1 ms, probably due to inertia. Therefore, the low resistance that was used before the resistance switch did not affect the slider signal. Then, as the slider speed increased, the sensing signal appeared, as shown using the red line. On the other hand, when there was no slider, the voltage was almost zero and flat, which makes the distinction between the two cases much easier.

### 6.4. Accuracy for Slider Proximity Detection

The detection method discussed in this work is intended to be used as a proximity sensor. In previous experiments, the slider was placed over the sensing electrode from the beginning of the experiments and, thus, did not show its performance as a proximity sensor. The following experiment verifies the performance of this sensing method in terms of proximity detection.

In the following experiment, the slider was placed a few centimeters away from the sensing electrode and was driven toward the sensing electrode with a pulse waveform frequency of 10 Hz, which moved the slider 40 steps per second. The voltage of the sensing electrode was continuously measured to reveal how the output grew as the slider approached. For the measurement, the resistance switching circuit in Figure 16 was utilized. As the circuit can be used only for the fourth voltage shift, the sensor output occurs once in four step movements, which corresponds to one cycle of the pulse waveform.

**Figure 18:**
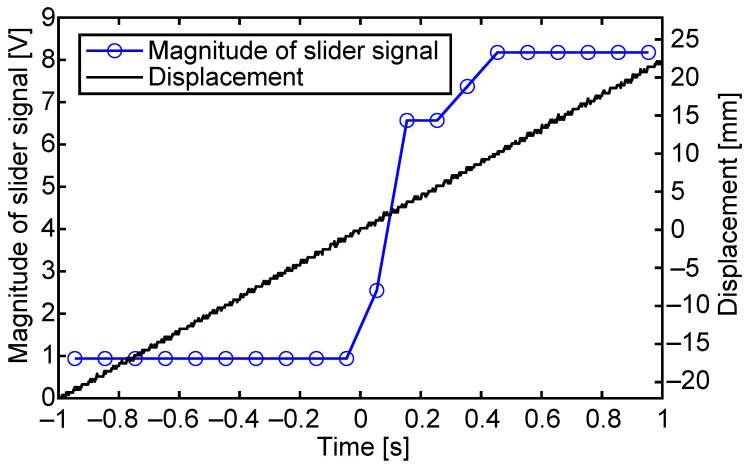
The magnitude of the slider signal when the slider is driven towards and over the sensing electrode. The edge of the slider reached the sensing electrode at 0 s.

Figure 18 shows the change in the magnitude of the slider signal while the slider was continuously moved toward the sensing electrode. Before 0 s, the slider did not cover the sensing electrode. At 0 s, the edge of the slider reached the sensing electrode, and after that, the slider moved above the sensing electrode. The signal magnitude was obtained from the maximum difference in the output voltages with and without the slider, in the period between 3 ms and 5 ms after the driving voltage shift.

In the plot, the sensing output rose in 2 cycles of the driving pulse waveform. In other words, the rising of the output signal is not very sharp. This is probably due to the fact that the charge distribution would not be perfect near the edge of the slider, and/or that the edge of the slider sheet would be slightly raised off of the stator surface, which decreases the capacitance coupling between the slider and the stator sensing electrode.

**Figure 19:**
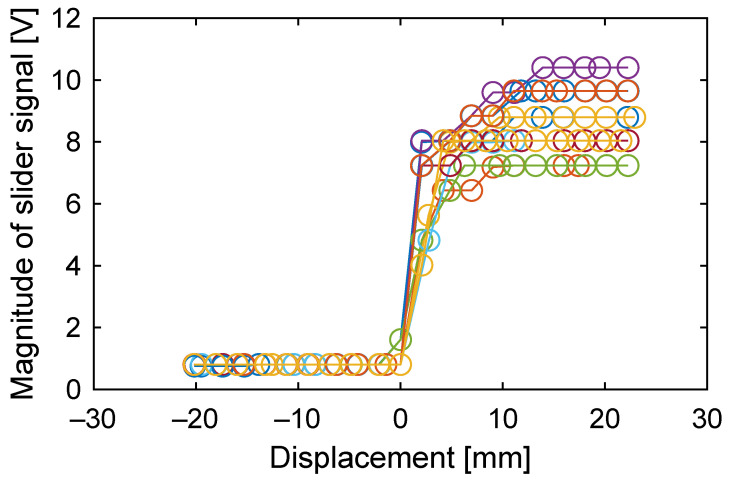
The magnitude of the slider signal against the slider displacement in ten measurements (the result of Figure 18 is not included). If a threshold is set at 2 V, for example, a slider proximity can be detected at the first data point above 2 V. All these data points appeared within 3 mm in these ten measurements.

In Figure 19, the magnitude of the slider signal is plotted against the slider displacement for ten different measurements to investigate the accuracy of the proximity detection. Since the output of the sensing circuit can be obtained once in one cycle of the pulse waveform, the slider moves 4 motion steps (2.4 mm maximum) between any two outputs. This theoretically limits the accuracy of the proximity detection; the accuracy cannot be lower than 2.4 mm. In the experiments, if we set a threshold at 2 V, the largest error found among the ten measurements was about 3 mm, which can be regarded as accuracy. In addition, the steady-state values were different between measurements. This is possibly due to the fluctuation of the air gap between the slider and the stator, as well as the changes in frictional conditions.

### 6.5. Discussions

In Figure 14, the experimental result showed a considerably similar output waveform to the analytical result. However, while there was good qualitative agreement, quantitative disagreement was found in the time constant of the voltage decay. Comparing our simulation using the proposed analytical model and the experimentally measured capacitances, the resulting time constant was found to be 1/3 of that observed in the experiment. In other words, the experimentally measured capacitances were smaller than expected.

This discrepancy is probably due to the capacitance of the cable, probe, and oscilloscope, as well as any other stray capacitance, connected to the sensing electrode. The effect of such capacitance, $C_{p}$, can be incorporated into the model by expanding the capacitance matrix as

(17) $$\begin{array}{cc} \tilde{C}\left(x\right)= & \left(\begin{array}{ccc} \tilde{T} & M(x) & \begin{array}{c} 0\\ -C_{p}\\ 0\\ 0 \end{array}\\ M^{t}\left(x\right) & L & \begin{array}{c} 0\\ 0\\ 0\\ 0 \end{array}\\ \begin{array}{cccc} 0 & -C_{p} & 0 & 0 \end{array} & \begin{array}{cccc} 0 & 0 & 0 & 0 \end{array} & C_{p} \end{array}\right) \end{array}$$

where $\tilde{T}$ is the same as T except for ${\tilde{T}}_{22}=4C_{0}-2C_{a}+\left(k+2\right)C_{t}+C_{p}$. By setting 70 pF to $C_{p}$, the analytical result showed a fairly consistent result with the experiment. Although we do not comprehend the exact input capacitance of the setup, 70 pF is within a reasonable range.

Another important point that should be discussed is the reliability of the measurement. As shown in Figure 15, the amplitude of the slider signal depends on the instantaneous speed of the slider. The slider speed can be affected by various factors, not just by the driving voltage amplitude. For example, if the friction between the stator and the slider changes, this will also change the instantaneous slider speed. If the environment is humid, this will decrease the surface resistance of the slider, which will shorten the length of the step movement and, thus, the speed. Or, even if the speed is maintained at the same level, the slider signal may decrease if the capacitance coupling between the slider and the stator is reduced. Such a reduction in the capacitance happens if the gap between the slider and the stator is enlarged, possibly due to curling of the sheet. The results in Figure 19 are a good example.

If the slider signal becomes too weak due to any of the above factors, a detection failure may occur. One good point, however, is that it works in a kind of fail-safe manner. Since the waveform only shows a strong slider signal when the actuator is properly operating, we can tell that a problem is happening in the actuator if the slider signal does not appear. Therefore, the detection method can also be used for health monitoring of the actuator system.

## 7. Conclusions

This paper discussed a method of detecting a dielectric slider sheet on a charge-induction type electrostatic actuator. The slider sheet is actuated by charge induction; a stable charge pattern is induced on the surface of the slide sheet, which is then propelled by electrostatic force from the stator parallel electrodes. The actuator is operated using pulse waveforms, and every time the voltage is shifted, the slider is moved one step. In the method discussed in this work, one of the stator electrodes is dedicated to sensing. When a slider with the induced charges moves over the sensing electrode, induction current flows in the sensing electrode, which is detected by the sensing circuit.

This paper proposed an analytical model and analytical procedure to predict the behavior of the sensing method. The model and procedure are based on a capacitance network model of the actuator. The results of the analysis indicated that the two different cases, with and without a slider over the sensing electrode, can be distinguished from the different shapes of their waveforms.

To facilitate robust sensing, this paper also proposed resistance switching. The resistance is first set to be small, such that the undesired impulsive output signal decays in a short time. Then, the resistance is switched to a large value, such that the target signal current can be amplified to a large output voltage. The analytical result clearly showed the benefit of the resistance switching. The output signal became distinguishable only with a simple threshold detection.

The detection method discussed in the analyses was experimentally verified using a prototype electrostatic actuator. Although quantitative discrepancies were found in the time constant of the voltage decay, likely due to the input capacitance of the measurement equipment, the experimentally observed waveforms matched quite well with the analytical results. It was confirmed in the experiment that the detection method can work as a proximity sensor, with an accuracy of about 3 millimeters.

## Figures and Tables

**Table 1:** Built-in sensing methods for electrostatic film actuators.

Actuator Type	Variable Capacitance		Charge Induction	
			(Low R Slider)	(High R Slider)
Ref.	[43]	[47]	[44]	This work
Principle	(*1)	(*2)	(*3)	(*4)
Electrode pitch	0.2 mm	0.2 mm	1 mm	0.6 mm
Max error	43 μm	∼0.2 mm	10–20 mm ^†^	

(*1): Measuring capacitance against slider electrodes using superposed high-f signal. (*2): Directly measuring driving current. (*3): Measuring capacitance against dielectric slider using superposed high-f signal. (*4): Measuring induced current caused by slider charges. ^†^: Estimated from the reported result.

## Data Availability

Experimental data are available upon request.
